# Budget-Constrained Edge Orchestration of DAG-Based Sensor-Data Workflows with Multicast Result Dissemination

**DOI:** 10.3390/s26196348

**Published:** 2026-10-08

**Authors:** Shijie Jia, Jianwei Zhang

**Affiliations:** School of Information Science and Engineering, Yunnan University, Kunming 650500, China; shijiej@stu.ynu.edu.cn

**Keywords:** sensor networks, edge computing, workflow scheduling, multicast communication, resource allocation

## Abstract

Distributed sensing applications increasingly transform observations through multi-stage pipelines executed across heterogeneous edge–cloud resources. A sensing request is operationally complete only after its actionable output reaches all subscribed monitoring or control endpoints. However, workflow scheduling and result dissemination are commonly optimized separately, even though the server executing the sink task determines the source of dissemination. This paper studies their coupling under a per-request budget. We model each sensor-data workflow as a directed acyclic graph of service-dependent processing stages and intermediate-data transfers, followed by shared delivery of the sink output. We formulate lexicographic minimization of end-to-end sensing-response delay and total cost, subject to precedence, server capacity, service availability, multicast connectivity, and budget constraints. We then propose Priority-Aware Constraint-based Large Neighborhood Search (PAC-LNS), which combines topology-aware prioritization, dynamic ready-window scheduling, bounded local refinement, dissemination-budget reservation, and shared-tree construction. We implement PAC-LNS scheduling and shared-tree construction in MATLAB and evaluate synthetic DAGs and Alibaba trace-derived computation workloads: feasibility determines admission, delay and cost are compared on common successful requests, and acceptance rate and online runtime are reported separately. In trace replay with R=5000, PAC-LNS accepts 89.04–90.12% of requests as the budget increases from B=100 to B=200, while its online scheduling and tree-construction time averages 16.644 ms per request across these settings. These results support joint processing and delivery orchestration for budget-constrained edge-assisted sensing services.

## 1. Introduction

Networked sensing systems support time-sensitive services such as industrial condition monitoring, environmental surveillance, mobile health, and intelligent transportation. A measurement is rarely the final product: observations may undergo filtering, feature extraction, multisensor fusion, inference, and decision generation before becoming actionable. Edge–cloud cooperation provides the heterogeneous computation needed by these pipelines but also makes stage placement and data movement integral to sensing-response latency [1,2,3].

This processing-to-action lifecycle differs from independent task offloading in two respects. First, the processing stages are linked by precedence and intermediate-data dependencies and may require services or models available on only a subset of servers. Second, the final detection, estimate, or decision may be consumed simultaneously by several subscribers, such as dashboards, alerting services, controllers, or actuators. A request, therefore, remains incomplete after the DAG sink finishes: its output must still reach all required endpoints. In an edge–cloud deployment, the placement of that sink determines the dissemination source and consequently changes both delivery delay and communication cost.

Recent sensor and Internet of Things (IoT) studies demonstrate the value of task-aware edge processing, cloud–edge collaboration, and service-chain orchestration [4,5,6]. Dependency-aware schedulers further consider DAG structure, heterogeneous resources, network flows, and service availability [7,8]. Learning-based schedulers, including graph-neural-network-assisted deep reinforcement learning and dependency-aware reinforcement learning, have also been investigated to capture complex DAG dependencies and adapt offloading decisions to dynamic edge conditions [9,10,11,12]. However, these approaches primarily optimize task placement or computation-stage completion and do not explicitly couple the selected result source with budget-feasible shared dissemination to multiple sensing endpoints. Conversely, multicast methods reduce duplicate transmissions to multiple receivers [13,14]. These lines of work largely stop at either computation completion or communication delivery. Optimizing them separately misses the placement-dependent transition from a computed sensing result to a delivered and actionable one.

We address this gap by jointly orchestrating a sensor-data DAG and the subsequent shared dissemination tree under a request budget. The scheduler assigns each processing stage to a compatible edge or cloud server; the server selected for the sink becomes the tree root. End-to-end delay is minimized first, while total processing and communication cost serves as a lexicographic secondary objective among delay-equivalent feasible solutions. PAC-LNS heuristically searches this feasible space without claiming global optimality; final admission uses a complete budget-feasible schedule–tree pair, and the evaluation separately measures delay and cost, acceptance, and decision time.

The main contributions of this paper are summarized as follows:

- We formulate the sensing request lifecycle as service-dependent DAG execution followed by placement-coupled multi-endpoint delivery, with a unified budget and end-to-end delay definition.
- We propose PAC-LNS, combining topology-aware priority construction, dynamic ready-window scheduling, dissemination-budget reservation, and bounded destroy-and-repair refinement.
- We construct a budget-feasible shared delivery tree that reuses common links and charges each tree edge once, connecting sink placement directly to dissemination delay and cost.
- We evaluate the orchestration layer on contrasting synthetic DAG topologies and Alibaba trace-derived workloads using delay, cost, acceptance rate, and online overhead.

The rest of this paper is organized as follows: Section 2 reviews related work; Section 3 presents the system model and problem definition; Section 4 details PAC-LNS; Section 5 evaluates performance; Section 6 discusses deployment and limitations; and Section 7 concludes the paper.

## 2. Related Work

### 2.1. Edge-Assisted Sensor-Data Processing

Edge processing moves latency-sensitive analytics closer to sensor gateways while retaining cloud capacity for compute-intensive stages. Recent work has considered task-aware fog/cloud scheduling for IoT logistics, age-efficient processing in multi-UAV sensor networks, cloud–edge scheduling for visual surveillance, and serverless or high-volume sensor-data analytics [2,3,4,5,15]. These systems show that sensing applications benefit from coordinated processing across heterogeneous tiers. Most, however, optimize task execution, freshness, or application-specific resource use without explicitly modeling shared delivery of the final result to multiple subscribers.

### 2.2. Dependency- and Service-Aware Workflow Orchestration

A multi-stage sensing pipeline can be represented as a DAG whose vertices are processing functions and whose edges carry intermediate data. Dependency-aware offloading methods optimize placement and execution while accounting for precedence, heterogeneous resources, or network flows [7,9,10,16]. Other studies consider startup delay, latency–cost tradeoffs, delay bounds, and dynamic service availability [17,18,19].

Limited edge storage and runtime capacity additionally prevent every node from hosting every service, model, or function image. Service caching and function-configuration research, therefore, couples executability with placement and configuration delay [6,8,20,21]. These approaches improve the computation stage but generally treat completion of the terminal task as completion of the request. They do not account for how the selected terminal server becomes the source of downstream result delivery.

### 2.3. Sensing-Result Dissemination in Edge Networks

Information-freshness scheduling coordinates sensing updates with processing in industrial systems [22], while multicast routing reduces redundant communication in smart sensor and edge networks [13,14,23]. In conventional multicast formulations, the source and receiver set are specified independently of application execution. In a processed sensing workflow, by contrast, the source does not exist at a fixed location in advance: it is the server selected for the sink task. This placement-dependent source is the key connection between workflow orchestration and shared dissemination addressed in this paper.

Table 1 highlights the unresolved intersection: sensor-oriented edge processing provides the application setting, while DAG scheduling, service availability, and multicast delivery are usually optimized in separate models. This work couples them through sink placement, budget reservation, and shared-tree dissemination.

## 3. System Model and Problem Definition

**Figure 1:**
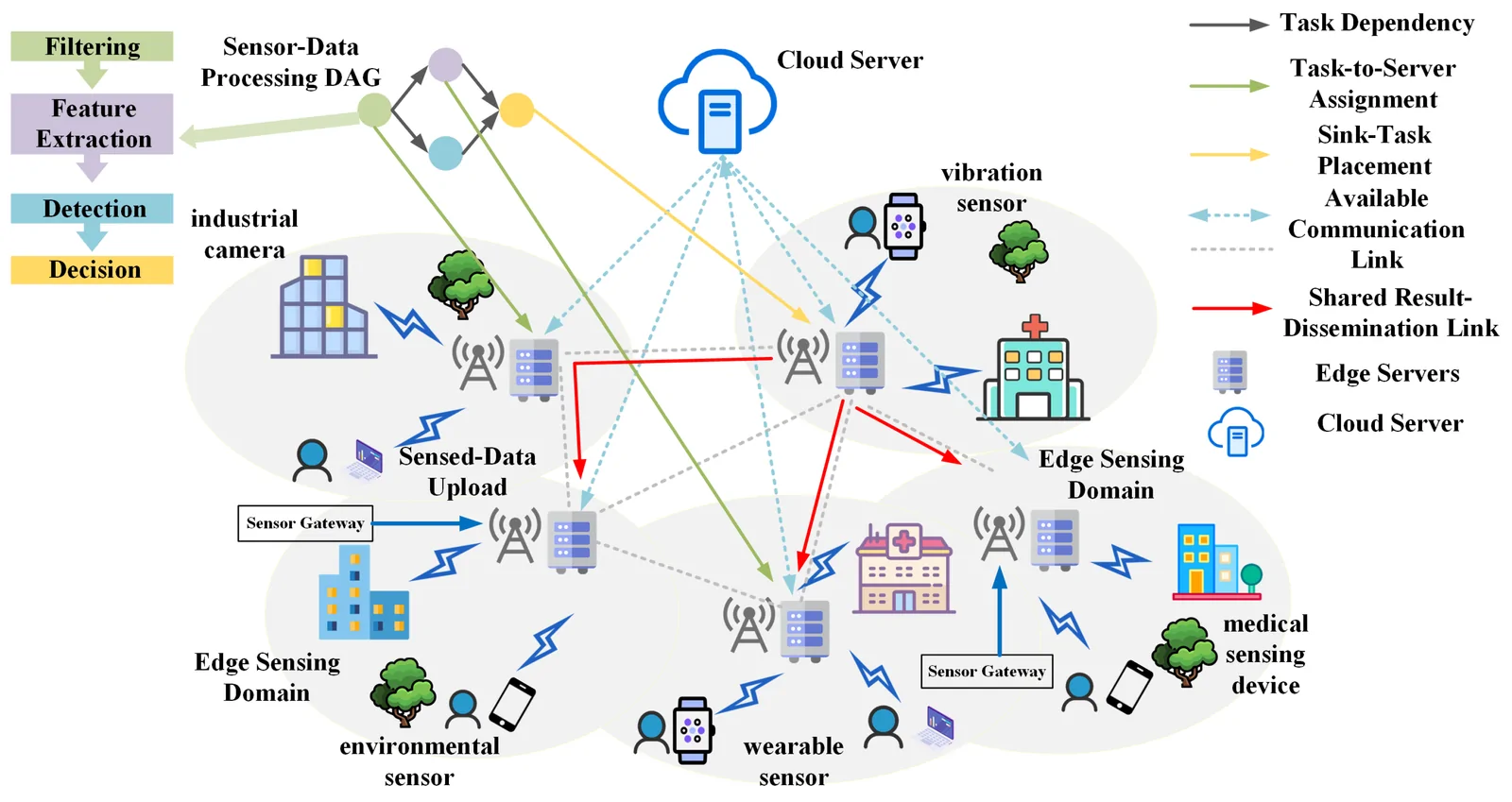
Edge-assisted sensor system with DAG-based processing and shared result dissemination to monitoring or control endpoints.

Figure 1 shows the complete request path considered in this paper. A sensor gateway converts an observation batch or detected event into a processing request. Its service-dependent stages execute across edge–cloud resources and exchange intermediate sensor-derived data. Completion of the sink stage creates an actionable output, such as a detection, estimate, or control decision, which is then delivered through a shared tree to every subscribed monitoring or control endpoint. The orchestrator therefore coordinates the interval from gateway submission to the last required result delivery.

For example, an industrial vibration event may trigger a workflow that performs signal filtering, frequency-domain transformation, feature fusion, and fault detection across nearby edge servers and the cloud. The resulting alarm is not useful until it reaches both the plant controller and the remote monitoring service; their delivery completion therefore belongs to the same end-to-end sensing request.

Table 2 summarizes the notation used repeatedly below; symbols local to a single equation or search step are defined at first use.

### 3.1. Edge–Cloud Sensor System and Workflow Model

The architecture comprises sensor gateways, distributed edge servers, and a centralized cloud. Gateways expose sensing requests to the orchestration layer, and heterogeneous servers execute their processing stages [24]. Let *S* be the server set and m=|S|. Edge servers offer low communication latency but limited capacity, whereas the cloud provides greater capacity with higher communication overhead [11].

For server j∈S, $C_{j}$ denotes concurrent resource capacity and $\rho_{j}$ denotes unit-time execution cost. For servers *u* and *v*, $c_{u,v}$ and $\sigma_{u,v}$ denote the transmission-delay and unit communication-cost coefficients, respectively [12].

A sensor-data processing request is modeled as a DAG [12,24], denoted by

(1) G=(V,E),

where *V* contains processing stages, such as filtering, fusion, feature extraction, detection, or inference, and *E* contains their precedence dependencies. A source vertex represents entry into the orchestrated pipeline rather than physical sensing, while a sink vertex marks production of the result to be disseminated. Let n=|V|. For task *i*, $t_{i,j}$ and $r_{i,j}$ denote its execution time and resource demand on server *j*. For (i,k)∈E, $a_{i,k}$ is the intermediate sensor-derived data volume; task *k* starts only after task *i* finishes and this data arrives.

Let $V^{\mathrm{real}}$ denote the set of physically executed processing tasks, and let

$$V^{\mathrm{exit}}=\left\{i\in V^{\mathrm{real}}|\mathrm{Succ}\left(i\right)\cap V^{\mathrm{real}}=⌀\right\}$$

denote the set of real exit tasks. Following the zero-work dummy-node convention commonly used to normalize multi-entry or multi-exit workflow DAGs [25], a virtual sink $v_{\mathrm{sink}}$ is appended as a common successor of all tasks in $V^{\mathrm{exit}}$. It is a logical completion barrier rather than a physical data-aggregation task:

$$F_{v_{\mathrm{sink}}}=S_{v_{\mathrm{sink}}}=\max_{i\in V^{\mathrm{exit}}}F_{i},\;e^{*}=\arg\max_{i\in V^{\mathrm{exit}}}F_{i},\;x_{v_{\mathrm{sink}}}=x_{e^{*}}.$$

Ties are resolved deterministically. The virtual sink and its incoming edges have zero execution time, resource demand, data volume, and cost; it only synchronizes completion and inherits $s=x_{e^{*}}$ as the dissemination source. For example, if two replicas of the same deterministic fault detector produce interchangeable alarms and both must finish, the last-finishing replica already holds a complete result for dissemination. If vibration and temperature branches instead produce complementary features, neither branch output is a complete diagnosis; a real fusion task must consume both, accounting for their transfers and execution before dissemination.

Because processing functions are deployed selectively, let $\Omega_{i}\subseteq S$ be the executable-server set of task *i*. Let $M_{i,j}=1$ when server *j* hosts task *i*’s required service and its placement is permitted and 0 otherwise. For real tasks, $\Omega_{i}=\left\{j\in S:M_{i,j}=1\right\}$; for zero-work virtual vertices, $\Omega_{i}=S$. Membership defines static eligibility; precedence, available capacity, and budget determine full feasibility. A valid assignment satisfies $x_{i}\in \Omega_{i}$, where $x_{i}$ is the selected server. Let $T_{0}$ and *B* denote request arrival time and budget, and let $S_{i}$ and $F_{i}$ denote task start and finish times. Thus,

(2) $$F_{i}=S_{i}+t_{i,x_{i}}.$$

The arrival time $T_{0}$ is the release time for entry tasks in Equation (3) and sets the time origin for the DAG makespan in Equation (12). The per-request budget *B* limits the combined computation and shared-tree dissemination cost in Equation (19); Equation (20) uses it provisionally during schedule construction.

After logical workflow completion at $v_{\mathrm{sink}}$, the request-level detection, estimation, or decision result of size $D_{f}$ is disseminated from the inherited source server $s=x_{e^{*}}$ to the active monitoring or control endpoints. Multicast and Steiner-tree techniques can reduce redundant delivery in edge networks [13,14]; the following subsections model the coupled scheduling and dissemination stages.

### 3.2. Sensor-Data Workflow Scheduling Model

The computation stage determines assignments and execution order under dependency, capacity, executability, and budget constraints. Following heterogeneous DAG scheduling [12], the earliest dependency-feasible start time of task *i* on server *j* is

(3) $$T_{i,j}^{\mathrm{dep}}=\left\{\begin{array}{cc} \max_{p\in \mathrm{Pred}(i)}\left(F_{p}+c_{x_{p},j}a_{p,i}\right), & \mathrm{Pred}(i)\neq ⌀,\\ T_{0}, & \mathrm{Pred}(i)=⌀, \end{array}\right.$$

where Pred(i) denotes the set of predecessor tasks of task *i* [26].

Even when dependency constraints are satisfied, task execution may still be delayed due to insufficient server resources. Let $S_{i,j}^{★}$ denote the earliest feasible start time of task *i* on server *j* under the resource-capacity constraint. The waiting time is then defined as

(4) $$W_{i,j}=\max\left\{0,\;S_{i,j}^{★}-T_{i,j}^{\mathrm{dep}}\right\}.$$

The computation-stage cost consists of execution cost and the communication cost required to transfer predecessor data. Following the joint delay–cost analysis commonly adopted in dependent task scheduling [27], the incremental cost of assigning task *i* to server *j* is defined as

(5) $$\Delta C_{i,j}=\rho_{j}t_{i,j}+\sum_{p\in \mathrm{Pred}(i)}\sigma_{x_{p},j}a_{p,i}.$$

The two terms in Equation (5) account for execution and predecessor-data transmission, respectively.

### 3.3. Shared Sensing-Result Dissemination Model

After the computation stage, the final sensing result available after logical workflow completion must be disseminated to multiple monitoring or control endpoints. Let $G_{N}=\left(S,L\right)$ denote the physical edge–cloud communication graph. The source server is $s=x_{e^{*}}\in S$, namely, the location inherited by the virtual sink from the last-finishing real exit task, and U⊆S denotes the set of active destination servers or gateways hosting the corresponding monitoring, alerting, or control services.

Instead of independently transmitting one copy of the result along a separate source–destination path for every destination, the considered dissemination stage constructs a shared multicast tree $T=(V_{T},E_{T})$ rooted at *s*, where $\left\{s\right\}\cup U\subseteq V_{T}$. A communication link shared by multiple destination paths appears only once in $E_{T}$ and therefore incurs transmission cost only once.

Because the delay coefficient $c_{u,v}$ and the communication-cost coefficient $\sigma_{u,v}$ may have different numerical scales, they are first normalized as

(6) $${\hat{c}}_{u,v}=\frac{c_{u,v}}{c_{\max}},\;{\hat{\sigma }}_{u,v}=\frac{\sigma_{u,v}}{\sigma_{\max}},$$

where $c_{\max}=\max_{(u,v)\in L}c_{u,v}$ and $\sigma_{\max}=\max_{(u,v)\in L}\sigma_{u,v}$.

For a tradeoff parameter α∈[0,1], the hybrid link weight is defined as

(7) $$w_{\alpha }\left(u,v\right)=\left(1-\alpha \right){\hat{c}}_{u,v}+\alpha {\hat{\sigma }}_{u,v}.$$

Thus, α=0 produces a delay-oriented link weight, whereas α=1 produces a cost-oriented link weight. A finite candidate set $W_{\alpha }$ is examined during tree construction to obtain alternative delay–cost tradeoffs.

For an attachment node $q\in V_{T}$ and an uncovered destination $v\in U\setminus V_{T}$, the candidate attachment path under α is defined as

(8) $$P_{\alpha }\left(q,v\right)=\arg\min_{P:q⇝v}\sum_{(u,k)\in P}w_{\alpha }\left(u,k\right).$$

For each partial tree, destination, and weight value, the attachment node is selected by

(9) $$q^{*}\left(T,v,\alpha \right)=\arg\min_{q\in V_{T}}d_{\alpha }^{\mathrm{sp}}\left(q,v\right),$$

where $d_{\alpha }^{\mathrm{sp}}\left(q,v\right)$ is the shortest-path distance induced by $w_{\alpha }$. The corresponding candidate path is $P_{\alpha }\left(q^{*},v\right)$; hence, one minimum-weight attachment is generated for each partial tree and each $\alpha \in W_{\alpha }$.

The selected path is attached to the current tree through an existing tree node. Only the previously unused links in $P_{\alpha }\left(q,v\right)\setminus E_{T}$ are added and charged. Candidate trees that exceed the remaining dissemination budget are discarded.

Let $D_{f}$ denote the size of the final result, and let $P_{T}\left(s,v\right)$ denote the unique root-to-destination path in the resulting multicast tree. Assuming that different branches can transmit in parallel, the completion delay of multicast dissemination is

(10) $$T_{\mathrm{mc}}=D_{f}\max_{v\in U}\sum_{\left(u,k\right)\in P_{T}\left(s,v\right)}c_{u,k}.$$

The multicast communication cost is evaluated over the set of unique tree links:

(11) $$C_{\mathrm{mc}}=D_{f}\sum_{\left(u,k\right)\in E_{T}}\sigma_{u,k}.$$

Consequently, a link shared by several root-to-destination paths is counted only once in $C_{\mathrm{mc}}$. This distinguishes the adopted shared-tree model from repeated unicast, in which the cost of a shared link would be charged separately for every destination.

### 3.4. Unified Delay–Cost Modeling

The zero-work virtual sink gives the computation-stage completion time:

(12) $$C_{\max}^{\mathrm{DAG}}=F_{v_{\mathrm{sink}}}-T_{0}=\max_{i\in V^{\mathrm{exit}}}F_{i}-T_{0},$$

which is the computation-stage makespan.

The end-to-end delay is defined as

(13) $$T_{e2e}=C_{\max}^{\mathrm{DAG}}+T_{\mathrm{mc}},$$

where $T_{\mathrm{mc}}$ denotes the delay of the multicast dissemination stage. Equation (13) therefore measures sensing-response time from gateway submission until the last subscribed endpoint receives the actionable result; DAG makespan alone would omit this final delivery interval.

The total system cost is defined as

(14) $$C_{\mathrm{tot}}=\sum_{i\in V^{\mathrm{real}}}\Delta C_{i,x_{i}}+C_{\mathrm{mc}},$$

Thus, Equation (14) accounts for both workflow execution and shared result delivery. The virtual source, virtual sink, and their zero-data logical edges add zero cost; consequently, only physically executed tasks and actual data-bearing transfers appear in the summation.

### 3.5. Feasibility Constraints and Problem Formulation

A feasible solution must satisfy task-executability, resource-capacity, precedence, and budget constraints. First, because heterogeneous edge nodes may only provide partial service or function support, each task must be assigned to an executable server:

(15) $$x_{i}\in \Omega_{i},\;\forall i\in V.$$

Second, for any server j∈S and any time instant t≥0, the resource-capacity constraint is given by

(16) $$\sum_{i\in A(j,t)}r_{i,j}\leq C_{j},$$

where

(17) $$A\left(j,t\right)=\left\{i\in V|x_{i}=j,\;S_{i}\leq t<F_{i}\right\}.$$

The schedule must also satisfy DAG precedence. For any task i∈V,

(18) $$S_{i}\geq \max_{p\in \mathrm{Pred}(i)}\left(F_{p}+c_{x_{p},x_{i}}a_{p,i}\right).$$

The actual budget feasibility of an accepted request must be evaluated using both computation expenditure and the communication cost of the constructed multicast tree. Using the real-task set $V^{\mathrm{real}}$ defined above, the exact end-to-end budget constraint is

(19) $$\sum_{i\in V^{\mathrm{real}}}\Delta C_{i,x_{i}}+C_{\mathrm{mc}}\left(T\right)\leq B,$$

where $C_{\mathrm{mc}}\left(T\right)$ is evaluated over the unique links of the shared multicast tree according to Equation (11).

During incremental schedule construction, however, the final multicast tree T is not yet available because its root depends on the server selected for the sink task. PAC-LNS, therefore, employs an estimated dissemination-budget reservation as an algorithmic feasibility guard. Let $C^{\mathrm{sch}}$ denote the accumulated computation cost of the tasks that have already been scheduled. When task *i* is tentatively assigned to server *j*, the scheduling-stage budget guard is defined as

(20) $$C^{\mathrm{sch}}+\Delta C_{i,j}+C_{\mathrm{res}}\left(i,j\right)\leq B.$$

The reservation term is defined as

(21) $$C_{\mathrm{res}}\left(i,j\right)=\left\{\begin{array}{cc} p_{r}\frac{{\bar{\delta }}_{j}}{\max\{1,d(i)\}},\; & d\left(i\right)\leq h_{r},\\ 0,\; & d\left(i\right)>h_{r}, \end{array}\right.$$

where $p_{r}$ is the reservation ratio, d(i) is the graph distance from task *i* to the sink task, $h_{r}$ is the sink-proximity threshold, and ${\bar{\delta }}_{j}$ is the estimated multicast cost when server *j* acts as the dissemination source. Let $Z_{j}=\left\{j\right\}\cup U$ contain the candidate source and all active destinations, and define their shortest-path communication-cost distance as

(22) $$d_{\sigma }^{\mathrm{sp}}\left(u,v\right)=\min_{P:u⇝v}\sum_{(a,b)\in P}\sigma_{a,b}.$$

Following the classical metric-closure approximation for the Steiner-tree problem [28], a complete metric-closure graph is constructed over $Z_{j}$ using $d_{\sigma }^{\mathrm{sp}}\left(u,v\right)$ as its edge weights. If $\mathrm{MST}(Z_{j})$ is a minimum spanning tree of this metric closure, the estimator used by the implementation is

(23) $${\bar{\delta }}_{j}=D_{f}\sum_{\left(u,v\right)\in \mathrm{MST}\left(Z_{j}\right)}d_{\sigma }^{\mathrm{sp}}\left(u,v\right).$$

This inexpensive estimator is used only by the incremental guard; final acceptance uses the exact unique-link cost in Equation (19). The defaults are $p_{r}=0.10$ and $h_{r}=1$. Because every real exit is one hop from the virtual sink and may determine the inherited source, $h_{r}=1$ reserves budget only when assigning such exits. Applying a source-dependent estimate to upstream tasks would unnecessarily restrict early decisions; the threshold is therefore structural rather than result-tuned.

Equation (20) is only an incremental guard; final acceptance still requires the exact joint budget in Equation (19) after tree construction.

Based on the above definitions, the optimization problem for each accepted sensor-data processing request is formulated as

(24) $$\begin{array}{cc} \underset{X,\;T}{\mathrm{lexmin}} & \left(T_{e2e},C_{\mathrm{tot}}\right)\\ \mathrm{s.t.} & (15),(16),(18),\\ \; & (19),\\ \; & E_{T}\subseteq L,\\ \; & \left\{s\right\}\cup U\subseteq V_{T},\\ \; & T\;\mathrm{is}\;\mathrm{connected}\;\mathrm{and}\;\mathrm{acyclic}. \end{array}$$

X=(x,S,F) denotes the computation schedule, including the task assignment, start-time, and finish-time decisions, and $T=(V_{T},E_{T})$ denotes the shared multicast tree rooted at the server executing the sink task. The computation schedule determines the dissemination source *s*, whereas the multicast tree determines the subsequent result-dissemination delay and communication cost. The objective is interpreted lexicographically: among solutions satisfying the exact budget and feasibility constraints, end-to-end delay is minimized first, and total cost is used as a secondary criterion when candidate solutions have the same end-to-end delay. Equation (24) defines the exact objective of the completed schedule–tree pair. During LNS, PAC-LNS uses the dissemination-aware proxy in Equation (47) to avoid repeatedly constructing a shared tree for every neighborhood candidate. The selected refined schedule is subsequently combined with the actual shared tree generated by the multicast-tree construction algorithm detailed in Section 4.5, where the exact objective and joint-budget feasibility are evaluated.

In addition to end-to-end sensing-response delay and total cost, the request-handling capability of the edge-assisted sensor system is evaluated using the acceptance rate:

(25) $$\mathrm{AR}=\frac{N_{\mathrm{acc}}}{N_{\mathrm{all}}},$$

where $N_{\mathrm{acc}}$ denotes the number of sensor-data processing requests satisfying the executability, precedence, capacity, and end-to-end budget constraints, and $N_{\mathrm{all}}$ denotes the total number of arriving sensing requests.

## 4. PAC-LNS Framework

### 4.1. Overall Algorithmic Architecture

PAC-LNS follows the same lifecycle as a sensing request: it first constructs and refines a feasible processing schedule, then uses the selected sink server as the root of shared result delivery. Unlike learning-based approaches such as DOS [12], its white-box decisions require neither offline training nor online neural inference. This makes the method suitable for a regional edge controller that must react to changing request arrivals while exposing the reason for each scheduling decision.

**Figure 2:**
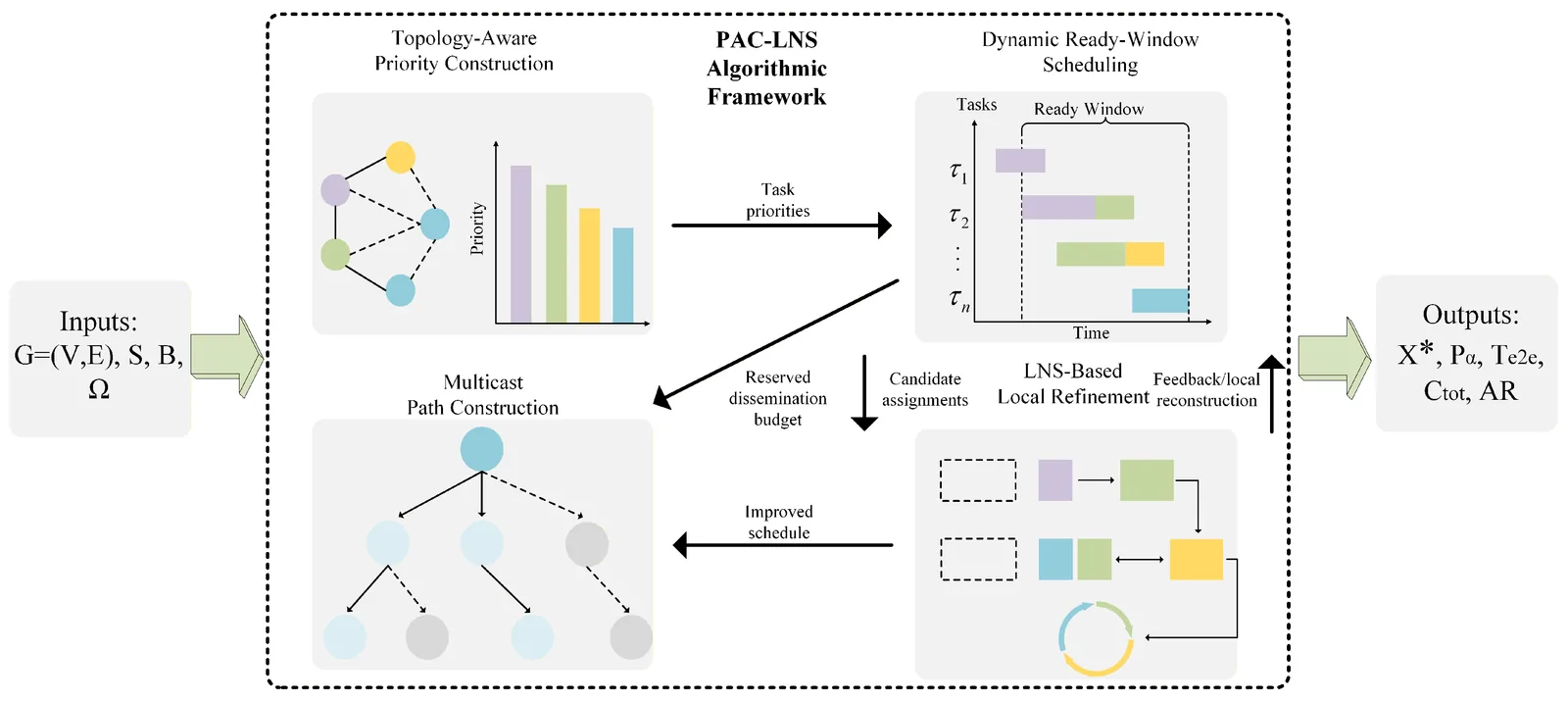
PAC-LNS framework for sensor-data workflow scheduling and shared result dissemination.

As shown in Figure 2, PAC-LNS constructs topology-aware priorities, generates a feasible schedule through dynamic ready-window selection, refines it using bounded LNS, and builds a budget-feasible shared tree rooted at the selected sink server. Algorithms 1–3 detail these phases.

### 4.2. Topology-Aware Priority Construction

To identify tasks that have a significant impact on the overall completion time, this paper adopts the priority-construction idea of HEFT [26]. Because service availability restricts task *i* to its executable-server set $\Omega_{i}$, its average execution time is calculated only over feasible execution locations:

(26) $${\bar{t}}_{i}=\frac{1}{|\Omega_{i}|}\sum_{j\in \Omega_{i}}t_{i,j},$$

where $\Omega_{i}\neq ⌀$; servers without the required service are excluded. For the positive-delay link set $L^{+}=\left\{\left(u,v\right)\in S\times S|c_{u,v}>0\right\}$, define

(27) $$\bar{c}=\left\{\begin{array}{cc} \frac{1}{|L^{+}|}\sum_{\left(u,v\right)\in L^{+}}c_{u,v},\; & L^{+}\neq ⌀,\\ 0, & L^{+}=⌀. \end{array}\right.$$

Based on the above definitions, the upward rank of task *i* is given by

(28) $${\mathrm{rank}}_{u}\left(i\right)=\left\{\begin{array}{cc} {\bar{t}}_{i}+\max_{k\in \mathrm{Succ}(i)}\left(\bar{c}\;a_{i,k}+{\mathrm{rank}}_{u}\left(k\right)\right), & \mathrm{Succ}(i)\neq ⌀,\\ {\bar{t}}_{i}, & \mathrm{Succ}(i)=⌀, \end{array}\right.$$

where Succ(i) is the successor set. Following HEFT [26], a larger rank indicates greater influence on the remaining critical path.

To further characterize the criticality of the remaining path after task *i* is finished, the tail critical-path length is defined as

(29) $${\mathrm{rank}}_{\mathrm{tail}}\left(i\right)=\max\left\{0,\;{\mathrm{rank}}_{u}\left(i\right)-{\bar{t}}_{i}\right\}.$$

Equation (29) removes the task’s own execution estimate and retains only unfinished downstream influence.

**Algorithm 1** Initial Scheduling Construction

***Input:*** *Sensor-data workflow* G=(V,E)*, server set S, budget limit B, parameters*  $\Theta =\{K,\beta ,\delta ,p_{r},h_{r}\}$***Output:*** *Initial schedule* X=(x,S,F)
  *1:* *Compute* ${\bar{t}}_{i}$*,* $\bar{c}$*,* ${\mathrm{rank}}_{u}\left(i\right)$*,* ${\mathrm{rank}}_{\mathrm{tail}}\left(i\right)$*, and* $P_{i}$  *2:* *Generate the global ordering* $\Pi^{\mathrm{PAC}}$  *3:* *Initialize* remaining←V*,* scheduled←⌀*,* C←0  *4:* ***while*** remaining≠⌀ ***do***  *5:*     *Generate the ready-task set* R(t)  *6:*     *Compute* $P_{i}^{\mathrm{dyn}}$ *for each* i∈R(t)  *7:*     *Select the top-K tasks to form* Q(t)  *8:*     best_pair←NULL  *9:*     ***for all*** i∈Q(t) ***do****10:*     ***for all*** $j\in \Omega_{i}$ ***do****11:*      *Compute* $T_{i,j}^{\mathrm{dep}}$*,* $S_{i,j}^{★}$*, and* $W_{i,j}$*12:*      *Compute* $F_{i}^{\left(j\right)}$*,* ${\hat{C}}_{i,j}^{\mathrm{cp}}$*, and* $\Gamma_{j}\left(S_{i}\right)$*13:*      *Compute* $\Delta C_{i,j}$ *and* ${\hat{T}}_{i,j}^{\mathrm{src}}$*14:*      ***if** capacity and dependency constraints hold **then****15:*       ***if*** $C+\Delta C_{i,j}+C_{\mathrm{res}}\left(i,j\right)\leq B$ ***then****14:*        *Update* best_pair *if* $z_{i,j}$ *is lexicographically smaller**17:*       ***end if****18:*      ***end if****19:*     ***end for****20:*     ***end for****21:*     ***if*** best_pair=NULL ***then****22:*      ***return** infeasible**23:*     ***end if****24:*     *Assign the selected task to the selected server**25:*     *Update* x*,* S*,* F*26:*     *Update* $C\leftarrow C+\Delta C_{i,j}$*27:*     *Move the scheduled task from* remaining *to* scheduled*28:* 
***end while***
*29:* ***return*** X=(x,S,F)



Traditional heuristic scheduling methods, such as HEFT, usually rely on a single upward-rank metric to prioritize tasks. However, in the edge–cloud collaborative environment considered in this paper, communication heterogeneity and constrained computing resources make a single metric insufficient for capturing the structural importance of tasks. To address this issue, PAC-LNS introduces a composite static priority score that integrates computation, communication, and topological aggregation features:

(30) $$P_{i}={\mathrm{rank}}_{u}\left(i\right)+\beta_{s}\phi_{i}+\beta_{c}\varphi_{i}+\beta_{p}\eta_{i},$$

where

(31) $$\phi_{i}=\frac{1}{\max\{1,d(i)\}},$$

(32) $$\varphi_{i}=\frac{\sum_{k\in \mathrm{Succ}\left(i\right)\cap V^{\mathrm{real}}}a_{i,k}}{\max\;\left\{\epsilon ,\max_{l\in V^{\mathrm{real}}}\sum_{k\in \mathrm{Succ}\left(l\right)\cap V^{\mathrm{real}}}a_{l,k}\right\}},$$

(33) $$\eta_{i}=\frac{|\mathrm{Pred}\left(i\right)\cap V^{\mathrm{real}}|}{\max\;\left\{1,\max_{l\in V^{\mathrm{real}}}\left|\mathrm{Pred}\left(l\right)\cap V^{\mathrm{real}}\right|\right\}},$$

Accordingly, the global initial ordering is obtained as

(34) $$\Pi^{\mathrm{PAC}}={\mathrm{argsort}}_{↓}\left(P_{i}\right).$$

In Equation (30), ${\mathrm{rank}}_{u}\left(i\right)$ captures the classical critical-path importance of task *i*, while $\beta_{s}$, $\beta_{c}$, and $\beta_{p}$ are weighting parameters. Their default values are $\left(\beta_{s},\beta_{c},\beta_{p}\right)=\left(0.55,0.25,0.20\right)$. Equation (31) defines the sink-proximity score as the reciprocal of the shortest graph distance from task *i* to the workflow sink, encouraging earlier production of the result to be disseminated. Equation (32) normalizes total outgoing data volume by its maximum over all real tasks, thereby identifying potential transfer hotspots. Equation (33) similarly normalizes the number of real predecessors and identifies synchronization barriers. Here, ϵ is a small positive constant used only to avoid division by zero. Descending order of the composite score converts the DAG into a priority-consistent sequence for subsequent scheduling decisions.

Algorithm 1 computes the priorities in Equations (26)–(34) and repeatedly evaluates ready-task–server pairs for dependency and capacity feasibility, reserved budget, completion estimate, pressure, and incremental cost. The best feasible pair is committed until all tasks are assigned, producing the starting point for LNS.

### 4.3. Dynamic Ready-Window Scheduling

Because the ready set changes during scheduling, PAC-LNS reorders R(t) at each scheduling instant *t* using the DAG-level normalizers

(35) $$\begin{array}{cc} r_{\mathrm{tail}}^{\max}\; & =\max\;\left\{\epsilon ,\max_{l\in V^{\mathrm{real}}}{\mathrm{rank}}_{\mathrm{tail}}\left(l\right)\right\},\\ \eta^{\max}\; & =\max\;\left\{1,\max_{l\in V^{\mathrm{real}}}\left|\mathrm{Pred}\left(l\right)\cap V^{\mathrm{real}}\right|\right\}. \end{array}$$

For any task i∈R(t), the dynamic priority is defined as

(36) $$\begin{array}{cc} P_{i}^{\mathrm{dyn}}= & \delta_{s}\frac{{\mathrm{rank}}_{\mathrm{tail}}\left(i\right)}{r_{\mathrm{tail}}^{\max}}+\delta_{c}\frac{|\mathrm{Pred}\left(i\right)\cap V^{\mathrm{real}}|}{\eta^{\max}}\\ \; & \;+\delta_{p}\frac{1}{\max\{1,d(i)\}}, \end{array}$$

where $\delta_{s}$, $\delta_{c}$, and $\delta_{p}$ are weighting coefficients. Their default values are $\left(\delta_{s},\delta_{c},\delta_{p}\right)=\left(0.70,0.20,0.10\right)$. These coefficients rank the ready tasks only; server assignment is subsequently determined by the lexicographic pair-selection rule below. The first two terms are normalized by DAG-level maxima computed over all real tasks rather than by maxima that vary with the current ready set. Equation (36) therefore provides a consistent priority scale across scheduling instants while emphasizing remaining criticality, synchronization complexity, and topological position.

Based on the dynamic priority values, the top-*K* tasks in the current ready-task set are selected to form the candidate task set:

(37) $$Q\left(t\right)=\mathrm{TopK}\left(R(t),K\right).$$

The candidate window limits detailed evaluation to *K* promising tasks.

Server assignment combines local completion with remaining critical-path influence:

(38) $${\hat{C}}_{i,j}^{\mathrm{cp}}=\left(F_{i}^{\left(j\right)}-T_{0}\right)+{\mathrm{rank}}_{\mathrm{tail}}\left(i\right),$$

where $F_{i}^{\left(j\right)}$ is the completion time on server *j*.

To prevent tasks from being continuously assigned to locally overloaded servers, the pressure of server *j* at time $S_{i}$ is further defined as

(39) $$\Gamma_{j}\left(S_{i}\right)=\frac{1}{C_{j}}\sum_{k:\;x_{k}=j,\;S_{k}\leq S_{i}<F_{k}}r_{k,j}.$$

A larger $\Gamma_{j}\left(S_{i}\right)$ indicates tighter concurrent resource pressure.

**Algorithm 2** Dissemination-Aware LNS Schedule Refinement
***Input:*** *Initial schedule* $X_{0}$*, iteration cap* $I_{\max}$*, maximum number of candidate neighborhoods* $K_{N}$*, neighborhood construction rule* Λ***Output:*** *Refined computation schedule* $X^{*}$ *for multicast-tree construction*
  *1:* $X^{*}\leftarrow X_{0}$*,* iter←0  *2:* ***while*** $\mathrm{iter}<I_{\max}$ ***do***  *3:*     *Compute* slack(i) *for each task*  *4:*     *Construct at most* $K_{N}$ *candidate neighborhoods* N *according to* Λ  *5:*     improved←FALSE  *6:*     ***for all*** R∈N ***do***  *7:*     *Release the server assignments of tasks in R*  *8:*     *Fix* $x_{i}=x_{i}^{*}$ *for* i∉R *and leave* i∈R *free*  *9:*     *Reorder R and form the repair priority seed**10:*     *Reset all task times and internal server intervals**11:*     *Rebuild a complete schedule using Algorithm 1 with the restricted server sets**12:*     *Obtain a candidate solution* $X^{\mathrm{new}}$*13:*     ***if*** $X^{\mathrm{new}}$ *satisfies Equations (15), (16), (18) and (20), and* $X^{\mathrm{new}}≻X^{*}$ ***then****14:*      $X^{*}\leftarrow X^{\mathrm{new}}$*15:*      improved←TRUE*16:*      ***break****17:*     ***end if****18:*     ***end for****19:*     ***if*** improved=FALSE ***then****20:*     ***break****21:*     ***end if****22:*     iter←iter+1*23:* 
***end while***
*24:* ***return*** $X^{*}$


For every feasible task–server pair (i,j), the dissemination-delay tie breaker is

(40) $${\hat{T}}_{i,j}^{\mathrm{src}}=\frac{D_{f}}{\max\{1,d(i)\}}\max_{v\in U}d_{c}^{\mathrm{sp}}\left(j,v\right),$$

where $d_{c}^{\mathrm{sp}}\left(j,v\right)$ is the delay-weighted shortest-path distance from candidate server *j* to destination *v*. PAC-LNS then forms the tuple

(41) $$z_{i,j}=\left({\hat{C}}_{i,j}^{\mathrm{cp}},F_{i}^{\left(j\right)},W_{i,j},{\hat{T}}_{i,j}^{\mathrm{src}},\Gamma_{j}\left(S_{i}\right),\Delta C_{i,j}\right)$$

and selects

(42) $$\left(i^{*},j^{*}\right)=\underset{i\in Q\left(t\right),\;j\in \Omega_{i}}{\arg\min_{\mathrm{lex}}}z_{i,j},$$

over the pairs satisfying service availability, precedence, capacity, and the budget guard in Equation (20). Thus, critical-path-aware completion is minimized first; task completion time, waiting time, the dissemination-delay proxy, server pressure, and incremental cost are applied successively as tie breakers.

### 4.4. LNS-Based Local Refinement

To escape local biases in the initial heuristic schedule, PAC-LNS repeatedly removes a bounded task subset and repairs it following the destroy-and-repair principle of LNS [29]. Only the server placements of unaffected tasks are retained; all task start and finish times are recomputed during repair. The neighborhood is schedule-dependent: critical or low-slack stages are selected first, their server assignments are released, and a complete candidate schedule is rebuilt in precedence-feasible order. Thus, refinement targets bottlenecks that delay result generation without reopening every server-placement decision.

More precisely, for a destroyed set *R*, the repair-stage candidate-server set is

(43) $$\Omega_{i}^{R}=\left\{\begin{array}{cc} \Omega_{i}, & i\in R,\\ \{x_{i}^{*}\}, & i\notin R, \end{array}\right.$$

Here, $x_{i}^{*}$ is the incumbent server. Reconstruction resets all task times and internal intervals but retains intervals of earlier accepted requests, then processes every task through the ready set. Hence, an unaffected successor waits for its repaired predecessor and recomputes both dependency-ready and capacity-feasible start times on its fixed server; no stale timing decision is retained.

To identify tasks that have a greater impact on the overall performance, the slack of task *i* is defined as

(44) $${\mathrm{slack}}_{X}\left(i\right)=\max\left\{0,\;C_{\max}^{\mathrm{DAG}}\left(X\right)-\left(F_{i}-T_{0}+{\mathrm{rank}}_{\mathrm{tail}}\left(i\right)\right)\right\}.$$

Here, $C_{\max}^{\mathrm{DAG}}\left(X\right)$ is the computation-stage makespan of the current solution. A smaller value indicates that the task lies closer to the critical region of the current schedule.

For a schedule *X*, let s(X) denote the server hosting the sink task. Before LNS begins, the shortest-path delay distance from every candidate source *s* to every active destination v∈U is precomputed as

(45) $$d_{c}^{\mathrm{sp}}\left(s,v\right)=\min_{P:s⇝v}\sum_{(u,k)\in P}c_{u,k}.$$

The dissemination-delay proxy used during LNS is then

(46) $${\hat{T}}_{\mathrm{mc}}\left(s\left(X\right)\right)=D_{f}\max_{v\in U}d_{c}^{\mathrm{sp}}\left(s\left(X\right),v\right).$$

This proxy measures the worst shortest-path delay from the candidate sink server to the destinations and avoids reconstructing a multicast tree for every LNS candidate. It allows LNS to distinguish two schedules with similar computation makespans but different result-source locations. The dissemination-aware schedule-evaluation function is defined as

(47) $$J\left(X\right)=C_{\max}^{\mathrm{DAG}}\left(X\right)+{\hat{T}}_{\mathrm{mc}}\left(s\left(X\right)\right).$$

The computation-stage cost associated with schedule *X* is

(48) $$C\left(X\right)=\sum_{i\in V^{\mathrm{real}}}\Delta C_{i,x_{i}},$$

where $\Delta C_{i,x_{i}}$ is given by Equation (5). It is used only as the secondary criterion when two candidate schedules have the same dissemination-aware delay value.

Within the LNS search, a reconstructed schedule replaces the current schedule if

(49) $$X^{\mathrm{new}}≻X^{\mathrm{old}}\Leftrightarrow \left\{\begin{array}{c} J\left(X^{\mathrm{new}}\right)<J\left(X^{\mathrm{old}}\right),\\ \mathrm{or}\\ J\left(X^{\mathrm{new}}\right)=J\left(X^{\mathrm{old}}\right)\\ \;\mathrm{and}\;C\left(X^{\mathrm{new}}\right)<C\left(X^{\mathrm{old}}\right). \end{array}\right.$$

This proxy ranks LNS candidates efficiently. After Algorithm 2 returns $X^{*}$, the exact remaining dissemination budget is

(50) $$B_{\mathrm{mc}}\left(X^{*}\right)=B-C\left(X^{*}\right).$$

The subsequent multicast-tree construction discards partial trees exceeding this budget and rejects the request if no complete tree remains; otherwise, Equations (13) and (14) give the exact metrics. Thus, the proxy guides search while tree construction certifies joint feasibility.

Four deterministic neighborhood families are generated from the current schedule. The critical-path family uses consecutive segments of the extracted critical path; the sink family groups tasks whose distance to the sink does not exceed two hops; the load family selects tasks from servers in descending order of accumulated execution load; and the slack family selects the tasks with the smallest values of ${\mathrm{slack}}_{X}\left(i\right)$. For Alibaba replay, the corresponding maximum destruction sizes are 6, 5, 4, and 4 tasks. Duplicate neighborhoods are removed and the remainder are ranked by critical-path membership and sink proximity; at most $K_{N}=3$ neighborhoods are tried per iteration. The selected tasks are reordered using normalized tail rank, predecessor aggregation, and sink proximity with weights (0.65,0.20,0.15), respectively. These repair-order weights are distinct from the dynamic ready-task weights used during initial construction. The tasks are then reconstructed using the feasibility checks of Algorithm 1. The synthetic tests retain the topology-dependent destruction scaling described in Section 5.1.4. Search terminates when no feasible lexicographic improvement is found or $I_{\max}$ is reached.

### 4.5. Multicast Path Construction

After the processing schedule has been obtained, the server hosting the sink task becomes the root of the result-dissemination tree. The procedure incrementally connects uncovered monitoring or control endpoints. For every partial tree, it considers multiple normalized delay–cost weights from $W_{\alpha }$ and evaluates attachment paths from existing tree nodes to the next destination. Only newly introduced links are added to the tree cost; links already used by previous destination paths are reused without duplicate charging.

To avoid committing prematurely to a single attachment decision, a bounded beam of candidate trees is maintained. Candidate trees violating the exact remaining dissemination budget in Equation (50) are removed, and delay–cost-dominated states are pruned. If the beam becomes empty, tree construction returns infeasible and the request is rejected; otherwise, the best surviving state is selected lexicographically by multicast completion delay and unique-link cost. This construction explicitly exploits link sharing and is therefore different from independently computing and charging one source–destination path for each receiver.

More precisely, a candidate tree $T_{a}$ dominates $T_{b}$ if

(51) $$T_{\mathrm{mc}}\left(T_{a}\right)\leq T_{\mathrm{mc}}\left(T_{b}\right),\;C_{\mathrm{mc}}\left(T_{a}\right)\leq C_{\mathrm{mc}}\left(T_{b}\right),$$

with at least one strict inequality. After infeasible and dominated states are removed, the survivors are sorted lexicographically by multicast completion delay first and unique-link cost second, and the first *H* states are retained. The default settings are H=3 and $W_{\alpha }=\left\{0,0.25,0.5,0.75,1\right\}$.

**Algorithm 3** Budget-Feasible Shared Multicast-Tree Construction
***Input:*** $G_{N}=\left(S,L\right)$*, s, U,* $D_{f}$*,* $B_{\mathrm{mc}}$*,* $W_{\alpha }$*, and H****Output:*** 
*Multicast tree $T^{*}$, $T_{\mathrm{mc}}$, $C_{\mathrm{mc}}$, and feasibility status $\phi_{\mathrm{mc}}$*

  *1:* *Normalize* $c_{u,v}$ *and* $\sigma_{u,v}$ *using Equation (6)*  *2:* *Construct* $w_{\alpha }\left(u,v\right)$ *for every* $\alpha \in W_{\alpha }$  *3:* 
*Order the destinations by nondecreasing delay-oriented distance from s*
  *4:* *Initialize beam* B *with the tree* $T_{0}=\left(\left\{s\right\},⌀\right)$  *5:* 
***for all** destinations v in the resulting order **do***
  *6:*     *Initialize candidate set* C←⌀  *7:*     ***for all*** T∈B ***do***  *8:*     ***if*** $v\in V_{T}$ ***then***  *9:*      *Add* T *to* C*10:*      ***continue****11:*     ***end if****12:*     ***for all*** $\alpha \in W_{\alpha }$ ***do****13:*      ***Select*** $q^{*}=\arg\min_{q\in V_{T}}d_{\alpha }^{\mathrm{sp}}\left(q,v\right)$*14:*      ***Compute*** $P_{\alpha }\left(q^{*},v\right)$*15:*      *Attach the unused links* $P_{\alpha }\left(q^{*},v\right)\setminus E_{T}$ *to obtain* $T^{\prime }$*16:*      *Update the root-to-node delays in* $T^{\prime }$*17:*      *Compute* $C_{\mathrm{mc}}\left(T^{\prime }\right)$ *over the unique links of* $T^{\prime }$*18:*      ***if*** $T^{\prime }$ *is acyclic and* $C_{\mathrm{mc}}\left(T^{\prime }\right)\leq B_{\mathrm{mc}}$ ***then****19:*        *Add* $T^{\prime }$ *to* C*20:*      ***end if****21:*     ***end for****22:*     ***end for****23:*     ***if*** C=⌀ ***then****24:*     $\phi_{\mathrm{mc}}\leftarrow \mathrm{FALSE}$*25:*     ***return** infeasible**26:*     ***end if****27:*     *Remove delay–cost-dominated trees from* C*28:*     *Retain at most the best H trees to form* B*29:* 
***end for***
*30:* *Select* $T^{*}\in B$ *lexicographically by multicast completion delay and unique-link cost**31:* *Compute* $T_{\mathrm{mc}}$ *using Equation (10)**32:* *Compute* $C_{\mathrm{mc}}$ *using Equation (11)**33:* 

$\phi_{\mathrm{mc}}\leftarrow \mathrm{TRUE}$

*34:* ***return*** $T^{*},T_{\mathrm{mc}},C_{\mathrm{mc}},\phi_{\mathrm{mc}}$


### 4.6. Algorithm Analysis

In this subsection, the proposed method is analyzed from three aspects, namely feasibility, finite termination, and time complexity.

**Property 1**  (End-to-end feasibility)**.** *Let $X^{*}$ be the refined schedule returned by Algorithm 2. If Algorithm 3 returns a complete tree $T^{*}$ under $B_{\mathrm{mc}}\left(X^{*}\right)=B-C\left(X^{*}\right)$, then the combined solution $\left(X^{*},T^{*}\right)$ satisfies the exact joint-budget constraint and all feasibility constraints in Equation (24).*

**Proof.**  Ready-set insertion and capacity checks establish schedule feasibility by induction; LNS rebuilds times using the same checks. Algorithm 3 roots a connected acyclic tree at $s(X^{*})$, covers *U*, and enforces $C_{\mathrm{mc}}\left(T^{*}\right)\leq B-C\left(X^{*}\right)$. The combined solution therefore satisfies Equation (24).    □

**Property 2**  (Finite termination)**.** *Each phase of PAC-LNS terminates after finitely many operations.*

**Proof.**  Algorithm 1 assigns one of *n* tasks per iteration or returns infeasible; LNS tests at most $I_{\max}K_{N}$ neighborhoods; and tree construction processes q=|U| destinations while retaining at most *H* states. All loops are therefore finitely bounded.    □

**Property 3**  (Time complexity)**.** *Under bounded implementation parameters, all three algorithms have polynomial time complexity.*

**Proof.**  Evaluating task–server pairs gives Algorithm 1 a worst-case complexity of $O(n^{2}m)$. Each LNS repair has the same asymptotic order, so Algorithm 2 is bounded by $O(I_{\max}K_{N}n^{2}m)$. Let $n_{\alpha }=\left|W_{\alpha }\right|$ and let $\chi_{\mathrm{sp}}$ be the cost of one shortest-path query. For each of the *q* destinations, Algorithm 3 examines at most *H* partial trees, $n_{\alpha }$ weights, and *m* attachment nodes; including dominance pruning, its bound is

(52) $$O\;\left(qHn_{\alpha }m\chi_{\mathrm{sp}}+q{\left(Hn_{\alpha }\right)}^{2}\right).$$

With Dijkstra’s algorithm, $\chi_{\mathrm{sp}}=O\left(|L|\log m\right)$. Since q≤m and the search parameters are bounded, the complete framework remains polynomial.    □

The dominant terms arise from schedule construction and LNS repair; tree construction adds the final term above.

## 5. Performance Evaluation

### 5.1. Experimental Setup

We evaluate the orchestration layer rather than a particular sensing algorithm. Topology-controlled DAGs isolate the effect of workflow structure, while trace-driven computation workloads test request admission, end-to-end delay, and online overhead under large-scale resource contention. Together, these experiments ask whether processing and delivery can be coordinated efficiently across sensing-pipeline structures and dynamic arrivals.

#### 5.1.1. Evaluation Datasets

*Synthetic DAG task graphs:* Fast Fourier Transform (FFT) and Gaussian Elimination (GE) workflows are adopted from scheduling benchmarks [30]. They are used as dependency-topology benchmarks, not as complete physical sensing applications. FFT represents parallel spectral-processing kernels common in vibration or acoustic analysis, whereas GE represents deeper asymmetric dependencies that can arise in estimation, reconstruction, and multisensor fusion. The budget is relaxed so that the results primarily expose scheduling behavior across these contrasting structures.

*Alibaba Cluster Trace:* Alibaba Cluster Trace v2018 [31] supplies production-derived computation-side DAG structures, durations, resource demands, and arrivals. It is used as a workload surrogate rather than represented as a sensor-measurement dataset. Following [12], we vary the replay scale from R=500 to 20,000 and impose budgets B=100–300 to examine cost, admission, and response QoS under resource contention.

#### 5.1.2. Trace-to-Workflow Mapping

We construct trace-driven dependent-task requests from the batch_task.csv table of Alibaba Cluster Trace v2018. After removing incomplete records, the remaining entries are grouped by job_name, and each group is treated as one DAG request. Following the encoding rule of the trace, the first numerical token in task_name identifies the current task, while the remaining numerical tokens identify its predecessor tasks. For example, M5_3_4 generates the dependency edges (3,5) and (4,5). Jobs containing invalid task identifiers, missing dependency endpoints, no dependency edge, or a cyclic reconstructed graph are discarded. A zero-work virtual source is connected to all entry tasks, and all real exit tasks are connected to a zero-work virtual sink through zero-data logical edges. These virtual vertices normalize the trace-derived DAG without physical execution or aggregation. The sink inherits the last real exit’s server; any required branch fusion must be represented by an explicit real task.

The original Alibaba timestamps are recorded in seconds. We apply a uniform time-compression mapping in which one trace second is interpreted as one simulation millisecond. Accordingly, the trace-derived base duration of task *i* is represented as max{end_time − start_time, 0.1} simulation milliseconds, and the same mapping is applied to request inter-arrival intervals. The core demand of task *i* is defined as max{1, ⌈plan_cpu/100⌉}. To emulate heterogeneous edge–cloud execution, the base duration is divided by a server-dependent speed factor sampled from [0.8,1.4] for edge servers and [2.0,2.5] for the cloud server. All extracted jobs are ordered by their trace timestamps. Using the fixed random seed 42, we select one starting position and construct nested continuous replay sequences containing R∈{500,1000,5000,10,000,20,000} requests. Arrival timestamps are shifted so that the first request arrives at time zero while the original inter-arrival intervals are preserved. For the R=5000 segment, 5000 requests span 1979 trace seconds, corresponding to 2.526 requests/s originally and 0.3959 simulation ms between requests on average after compression (approximately 2526 requests per simulation second). Applying the same factor to task durations and inter-arrival intervals preserves their relative timing, order, and burst structure. Because Alibaba lacks network measurements, communication is introduced directly in simulation milliseconds using Table 3; the replay is therefore a contention stress test rather than a claim of physical computation–communication calibration or real-time controller throughput. All methods share the same replay and network realization.

Because the trace lacks the network and sensing-service attributes required by the model, service compatibility, data volumes, subscribers, result sizes, and budgets are generated as controlled parameters. Approximately 40% of task–edge execution options are masked, the cloud supports all task types, and 70% of edge servers act as result destinations. Dependency volumes are sampled from [10,30] MB; final-result sizes are bounded by [5,500] MB and scaled with DAG size and dependency data. The replay therefore preserves trace-derived DAGs, durations, CPU demands, and arrivals while testing orchestration behavior rather than sensing accuracy or signal quality.

#### 5.1.3. Workload and Environment Configuration

All experiments were conducted in MATLAB R2024b on a 64-bit Microsoft Windows system with an Intel Core Ultra 5 125H processor (3.60 GHz as reported by the operating system) and 24 GB RAM. The request loop was executed serially without a parallel pool or GPU acceleration.

Table 3 gives the millisecond-scale simulator settings. With seed 42, 30% of edge servers (at least one) are sampled as strong; link coefficients are sampled per direction, averaged to symmetric matrices, and set to zero on the diagonal. For each real task, edge compatibility is sampled independently with probability 0.6; non-entry tasks are cloud-executable, entry tasks are restricted to edge servers, and destinations are sampled uniformly without replacement. These normalized settings model a faster but costlier and communication-distant cloud [32,33], not a specific provider.

**Table 3:** Main simulation parameters.

Parameter	Setting
Infrastructure	9 edge servers and 1 cloud server; capacities 8 and 10,000 cores
Processing rate	Normal edge: U(700,900); strong edge: U(1000,1400); cloud: 5000 MI/ms
Execution cost	Edge: 0.15–0.30; cloud: 0.55 units/ms
Synthetic task attributes	Workload U(5000,20,000) MI; cores uniform on {1,2,3,4}; data U(10,30) MB
Link delay	Edge–edge: U(0.02,0.05); cloud–edge: U(5.30,5.60) ms/MB
Link cost	Edge–edge: U(0.01,0.05); cloud–edge: U(5.30,5.60) units/MB
Availability mask	Approx. 40% of edge options
Destinations; result size	Synthetic: 50–70%; Alibaba: 70%; 5–500 MB
Budget; request scale	B=100–300; R=500–20,000
Synthetic scale	FFT: N=4–64; GE: N=5–30
Priority weights	Static: (0.55,0.25,0.20); dynamic: (0.70,0.20,0.10); repair: (0.65,0.20,0.15)
Search controls	K=4, $I_{\max}=10$, $K_{N}=3$, H=3
Reservation; α set	$\left(p_{r},h_{r}\right)=\left(0.10,1\right)$; $W_{\alpha }=\left\{0,0.25,0.5,0.75,1\right\}$

Table 3 summarizes the controlled scenario settings of the millisecond-granularity discrete-event simulator. The one-cloud, nine-edge architecture uses heterogeneous capacities; normalized execution cost increases with computing capability, and cloud–edge communication incurs higher delay and cost than edge–edge communication. Service masking represents deployment restrictions, while dataset-specific destination settings are reported in the table and the trace-mapping description. These choices follow delay–cost modeling principles for heterogeneous edge–cloud and cyber–physical workflow scheduling without relying on provider-specific prices [32,33].

#### 5.1.4. Evaluation Protocol and Baselines

We compare PAC-LNS with PACP-HEFT [24], an online priority-adjusted HEFT scheduler, and TUL [34], an offline approximation method for dependent task offloading with service caching. All methods use the same infrastructure, request sequence, and shared multicast-tree module. A request is counted as accepted only when both its computation schedule and complete multicast tree are feasible and their exact joint cost does not exceed the request budget. Acceptance rate is calculated over all arriving requests. When admission decisions differ, end-to-end delay and total cost are evaluated on the common successful set so that all methods are compared on identical requests. Runtime is wall-clock time measured around the online replay loop and includes scheduling, LNS refinement where applicable, multicast-tree construction, feasibility checks, and minor per-request state updates; trace loading and DAG-template preprocessing are excluded. Simulated E2E delay, $\left(F_{v_{\mathrm{sink}}}-T_{0}\right)+T_{\mathrm{mc}}$, includes resource waiting, execution, intermediate-data transmission, and dissemination, but excludes controller wall-clock computation, controller-side queueing, signaling, and rule-installation delays.

All stochastic scenario generation and replay sampling use the fixed seed 42, and each compared method starts from the same empty system state. The reported results, therefore, describe one controlled, reproducible realization rather than confidence intervals across independent seeds. For Alibaba replay, the fixed ready-window and neighborhood settings in Table 3 handle the large number of small DAG-like requests. The larger synthetic FFT and GE graphs instead use topology-dependent adaptation: the ready window is restricted for highly parallel FFT graphs to avoid excessive dispersion, whereas the destruction scale grows with GE graph size so that approximately 10–15% of their tasks can be reconstructed.

In every case, adaptation changes only the bounded search effort; the feasibility constraints, objective, infrastructure, and evaluation metrics remain unchanged. The search remains capped by $I_{\max}$ and $K_{N}$.

### 5.2. Evaluation on Synthetic DAG Task Graphs

This subsection evaluates PAC-LNS on two representative synthetic DAG task graphs, namely FFT and GE, under a relaxed-budget setting. The purpose is to examine the scheduling quality and scalability of the compared methods under different DAG topologies, without making the results dominated by budget-induced request rejection.

#### 5.2.1. Results on FFT Task Graphs

**Figure 3:**
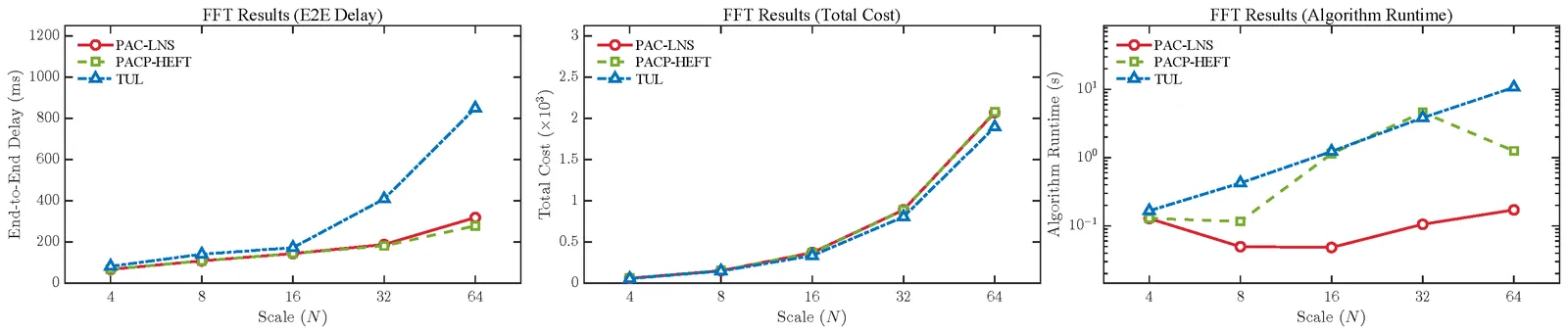
Performance comparison on FFT synthetic DAG task graphs in terms of end-to-end delay, total cost, and algorithm runtime.

As shown in Figure 3, PAC-LNS achieves the lowest runtime across all tested scales while maintaining delay and cost close to PACP-HEFT and superior to TUL-MPH. This efficiency stems from topology-aware priority construction, dynamic candidate-window selection, and bounded local refinement. The limited delay margin on FFT is expected because priority-based heuristics already perform well on its regular parallel structure. For sensing pipelines dominated by parallel spectral stages, the principal benefit is therefore low orchestration overhead rather than a large latency reduction.

#### 5.2.2. Results on GE Task Graphs

**Figure 4:**
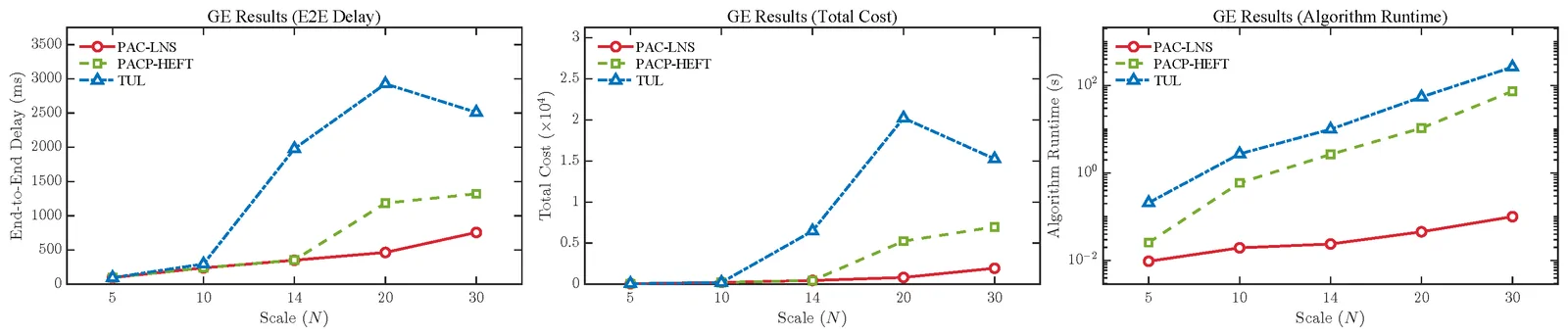
Performance comparison on GE synthetic DAG task graphs in terms of end-to-end delay, total cost, and algorithm runtime.

As shown in Figure 4, PAC-LNS achieves lower delay and slower cost growth on the deeper and more asymmetric GE graph, with a larger advantage for larger graphs. Critical-path and low-slack reconstruction is more effective because scheduling errors accumulate along long dependency chains. PAC-LNS also retains the lowest decision overhead. This result indicates that bounded refinement is most useful for sensing workflows containing serial estimation, fusion, or decision dependencies.

### 5.3. Trace-Driven Orchestration Evaluation

#### 5.3.1. Acceptance Rate Under Budget Constraints

**Figure 5:**
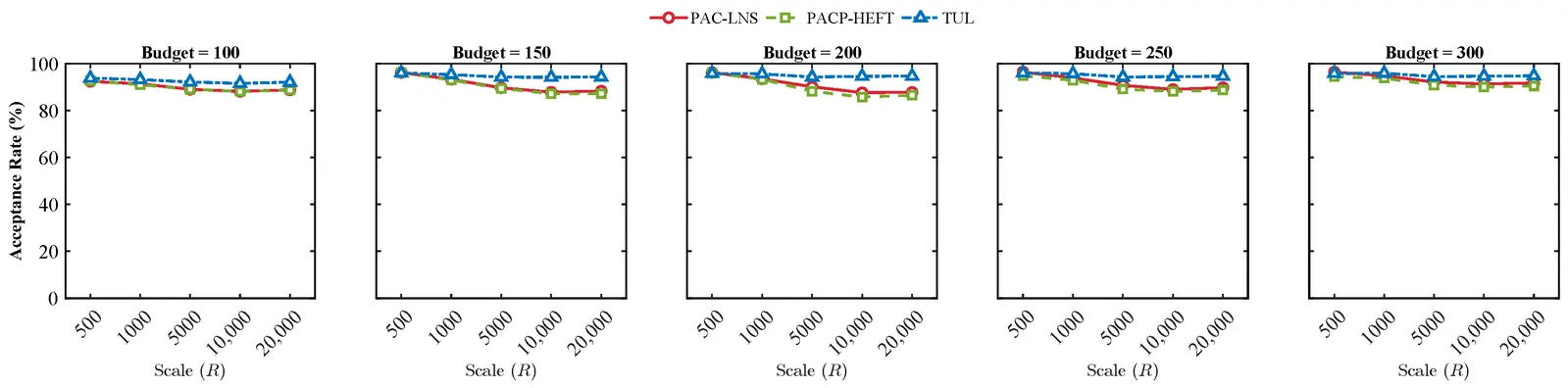
System acceptance rate under different budget settings and workload scales on Alibaba Cluster Trace.

Figure 5 shows that PAC-LNS is competitive with PACP-HEFT and usually admits more requests, whereas TUL-MPH usually has the highest acceptance but substantially longer common-set E2E delay. Final admission always uses exact tree and joint-budget feasibility; Section 5.3.4 isolates the budget-dependent reservation effect.

#### 5.3.2. QoS Analysis on the Common Successful Set

**Figure 6:**
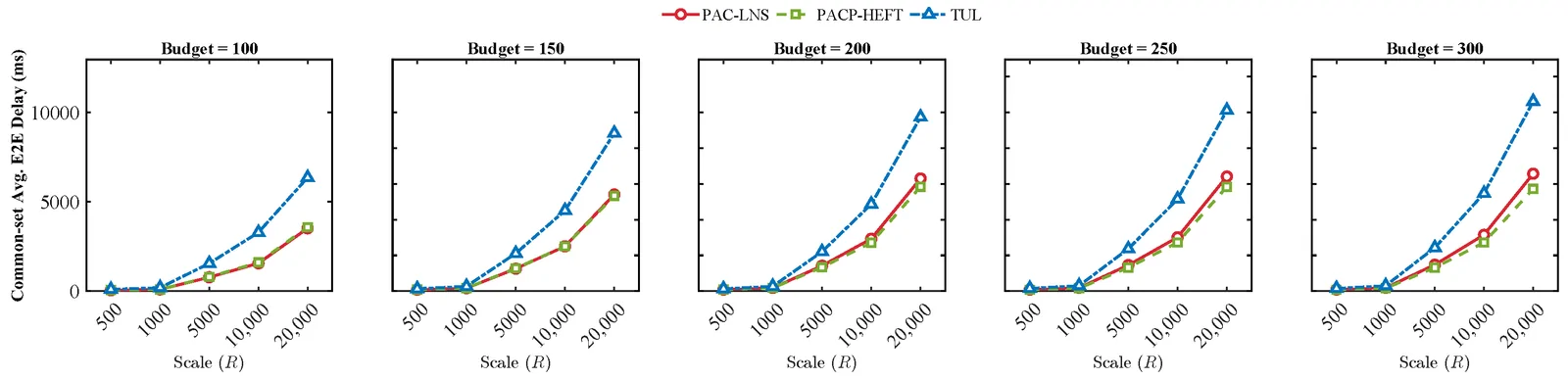
Average end-to-end delay on the common successful set under different budget settings and workload scales on Alibaba Cluster Trace.

To avoid statistical bias caused by different acceptance rates, Figure 6 reports the average end-to-end delay only on the common set of workflow instances successfully scheduled by all compared methods. On requests accepted by all methods, PAC-LNS remains close to PACP-HEFT and outperforms TUL-MPH at large scales. This comparison removes selection bias caused by different acceptance rates and shows that admitting more sensing requests does not require a substantial increase in time to result for the common workload.

#### 5.3.3. Algorithm Overhead and Scalability

**Figure 7:**
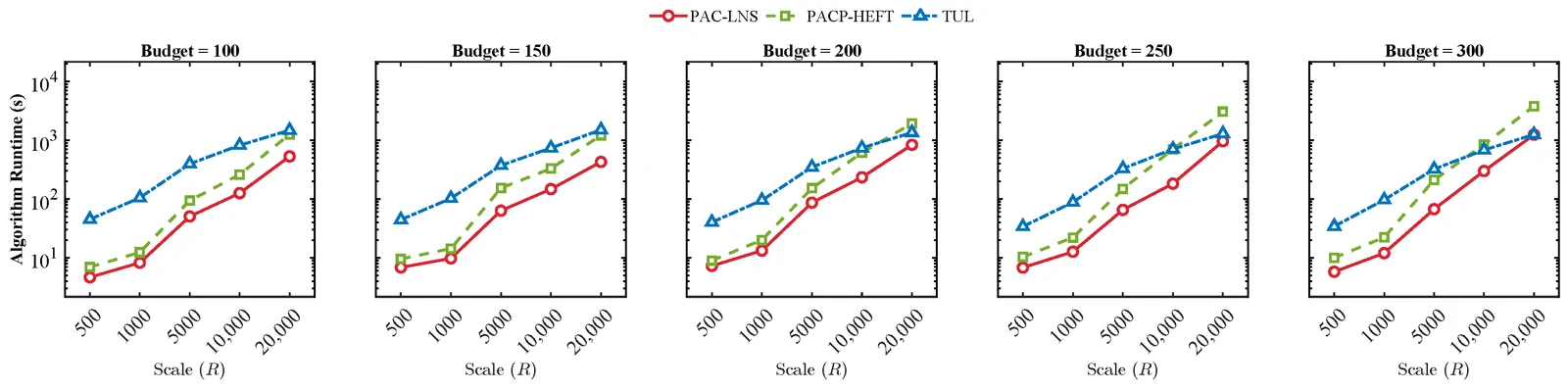
Algorithm runtime under different budget settings and workload scales on Alibaba Cluster Trace, where the vertical axis is shown on a logarithmic scale.

Figure 7 compares algorithm runtime under different budget settings and workload scales. PAC-LNS has the lowest runtime across all tested budget–scale settings, consistent with its restricted ready window and bounded reconstruction budget. Because this time is incurred before task dispatch, low decision overhead is important when gateways generate frequent sensing requests.

The 16.644 ms/request ablation runtime corresponds to 60.1 serial decisions/s. This exceeds the original 2.526 requests/s trace rate but not the compressed 2526 requests/s, so compressed replay is a contention stress test rather than a real-time throughput demonstration.

#### 5.3.4. Ablation Study

At R=5000 and B∈{100,150,200}, four variants replace PAC priority, set K=1, disable LNS, or set $p_{r}=0$. Other settings and the empty initial state are shared. Acceptance uses all requests; delay and cost use common sets of 4387, 4360, and 4347 requests. Runtime excludes template preprocessing.

Table 4 and Table 5 show modest, budget-dependent effects. PAC priority changes acceptance by at most 0.10 points and lowers delay at B=100,150; the full ready window lowers delay at these budgets and, at B=200, raises acceptance by 1.34 points and lowers cost by 2.22% versus K=1. LNS lowers delay by 0.15–1.27% with at most a 0.20-point admission change. Reservation raises acceptance by 0.16 and 0.90 points at B=100,150, ties at B=200, and lowers cost at the two larger budgets; it is protective, not uniformly superior. The full setting balances quality and its 16.644 ms overhead.

#### 5.3.5. Parameter Sensitivity

Table 6 varies one factor at R=5000, B=150; all others are fixed. The λ test scales only static priority. Acceptance uses all requests, whereas delay and cost use a family-specific common set and are comparable only within a block.

Quality varies little but exposes tradeoffs. K=2 minimizes delay, while default K=4 minimizes cost; larger windows add time without consistent gain. Versus $p_{r}=0$, default $p_{r}=0.10$ raises acceptance by 0.90 points and lowers cost by 0.97%, with 0.54% higher delay. LNS adds 0.20 admission points and lowers delay/cost by 0.14%/0.93%, saturating by $I_{\max}=5$. Scaling static weights leaves this replay unchanged. The coarse α set matches the five-weight default in quality while reducing runtime by 11.2%.

For H∈{1,2,3,5,10} at R=5000, B=150, feasibility and common-set multicast quality are identical (2.03 ms delay; 4.24 cost), while tree-construction time rises from 1.8889 ms/call at H=1 to 3.6938 at default H=3 and 9.6943 at H=10. Thus, quality saturates early; H=3 limits premature pruning at moderate cost, while H=1 is a faster option on similar small topologies.

#### 5.3.6. Robustness to Dissemination Attributes

Table 7 reports the sensitivity of acceptance rate and common-set end-to-end delay to generated dissemination attributes at R=5000 and B=150. We vary the destination ratio over {0.3,0.5,0.7,0.9} using nested destination sets. At the baseline ratio of 0.7, result sizes $D_{r}^{\left(0\right)}$ are compared with $D_{r}^{\left(0\right)}U_{r}$, where $U_{r}∼U\left(0.5,1.5\right)$, and $D_{r}^{\left(0\right)}e^{Z_{r}}$, where $Z_{r}∼N\left(-0.18,0.6^{2}\right)$; all sizes are truncated to [5,500] MB. All methods use the same seed-42 samples.

Across destination ratios, PAC-LNS acceptance changes by only 0.42 points, while multicast delay/cost rises from 1.877/2.373 to 2.138/5.589; shared links and computation-dominated E2E delay moderate the impact. Its E2E delay remains 40.2–40.5% below TUL-MPH, which has higher acceptance and lower cost. Across size models, PAC-LNS acceptance stays at 89.68–90.00% and E2E variation within 1.63%. PACP-HEFT is slightly faster in E2E under randomized sizes, whereas PAC-LNS retains higher acceptance and lower online runtime. The main balance is therefore robust without uniform metric dominance.

## 6. Discussion

### 6.1. Deployment Considerations

PAC-LNS can run at a regional edge controller or cloud control plane. A sensor gateway submits a workflow identifier, budget, subscriber set, and resource profile when an observation batch or event triggers processing. A workflow catalog supplies DAG structure and service requirements, while service registration and infrastructure monitoring provide function availability and server state. The controller dispatches each stage when its dependencies and intermediate data are ready; after sink completion, it installs a shared tree that delivers the output to monitoring, alerting, or control services.

The controller requires $O(m^{2})$ memory for link matrices and O(|V|+|E|) state per DAG; its output comprises O(|V|) assignments and at most $O(|E_{T}|)$ tree links. The measured runtime supports online use at the evaluated scales but excludes monitoring, control-message delivery, and rule-installation delays. Relative to the original trace rate, this is practical per-request overhead; however, it does not establish that one serial MATLAB controller can ingest the time-compressed replay in real time.

### 6.2. Limitations

The simulation assumes centralized, consistent state information and controlled, time-invariant links; stale monitoring, failures, packet loss, transient congestion, and competing multicasts are not modeled. The study begins at gateway submission and evaluates computation and communication orchestration, not transduction, measurement noise, sensing accuracy, sampling quality, or device energy. Alibaba supplies production-derived DAGs, durations, CPU demands, and arrivals, but service availability, communication parameters, subscribers, result sizes, and budgets are controlled; replay also uses time compression. Controller-side decision queueing is not modeled; sustaining the compressed stream in real time would require parallel or batched control, compiled implementation, or a less aggressive compression factor. All stochastic scenario parameters use the fixed seed 42, so the reported values describe one reproducible realization rather than confidence bounds. These choices support controlled algorithm comparison but limit statistical and operational generalization. Future work will consider multi-seed evaluation, time-varying networks, distributed scheduling, and sensor-network testbed validation.

## 7. Conclusions

This paper treated a sensing request as complete only when its DAG-generated result reaches every required monitoring or control endpoint. On this basis, we formulated budget-constrained edge–cloud orchestration that couples service-dependent processing with placement-dependent shared dissemination. PAC-LNS combines topology-aware scheduling, bounded refinement, delivery-budget reservation, and shared-tree construction. Synthetic and trace-driven experiments show that PAC-LNS provides a competitive balance among request acceptance, end-to-end delay, exact joint-budget feasibility, and low online overhead under the evaluated settings. The results establish a reproducible orchestration-layer baseline; future work will integrate time-varying networks, sensing-quality requirements, and physical sensor-system validation.

## Figures and Tables

**Table 1:** Comparison of representative related studies.

Refs.	Task Dependency	Service Availability	Multicast Dissemination	Current Limitations and Our Highlights
[2,3,4,5,15]	Partial	Partial	No	Sensor-oriented edge processing is studied, but shared delivery from a placement-dependent workflow sink is not modeled.
[7,8,9,10,16]	Yes	Partial	No	Computation-stage scheduling is emphasized, while result dissemination is not explicitly modeled.
[6,18,19,21]	Partial	Yes	No	Service availability is modeled, but its cross-stage impact on result dissemination is rarely considered.
[13,14,23]	No	Partial	Yes	Dissemination is optimized, but general DAG execution with precedence and synchronization constraints is not addressed.
**This work**	Yes	Yes	Yes	Simulation-based evaluation with centralized orchestration, controlled network parameters, and task-level trace abstraction.

**Table 2:** Principal notation used in the model and algorithm.

Symbol	Meaning
G=(V,E)	Workflow DAG: tasks and precedence edges
$V^{\mathrm{real}},V^{\mathrm{exit}},v_{\mathrm{sink}}$	Real tasks, real exit tasks, and virtual sink
$G_{N}=\left(S,L\right)$	Edge–cloud graph: servers and communication links
$M_{i,j},\Omega_{i},x_{i}$	Service/placement mask, executable-server set, and assignment of task *i*
$T_{0},B$	Request arrival time and total budget
$t_{i,j},r_{i,j},C_{j}$	Execution time, resource demand, and server capacity
$S_{i},F_{i}$	Start and finish times of task *i*
$a_{i,k},c_{u,v},\sigma_{u,v}$	Intermediate-data volume, link-delay coefficient, and link-cost coefficient
$\rho_{j}$	Unit-time execution cost on server *j*
$s,U,D_{f}$	Dissemination source, destinations, and result size
$T=(V_{T},E_{T})$	Shared dissemination tree and its nodes and links
$T_{e2e},C_{\mathrm{tot}}$	End-to-end delay and total cost
$C^{\mathrm{sch}},C_{\mathrm{res}},{\bar{\delta }}_{j}$	Accumulated computation cost, reservation, and estimated multicast cost
$K,I_{\max},H,\alpha$	Ready-window size, LNS limit, beam width, and link-weight tradeoff

**Table 4:** Ablation results on Alibaba trace replay (R=5000). E2E delay is calculated on the common successful request set.

Variant	*B* = 100		*B* = 150		*B* = 200	
	AR (%)	E2E (ms)	AR (%)	E2E (ms)	AR (%)	E2E (ms)
Full PAC–LNS	89.04	773.55	89.68	1252.26	90.12	1418.86
w/o PAC priority	89.08	776.44	89.58	1261.85	90.16	1400.54
K=1	89.12	785.96	89.64	1261.92	88.78	1414.85
w/o LNS	89.16	782.70	89.48	1254.10	90.18	1437.12
w/o budget reservation	88.88	777.21	88.78	1245.54	90.12	1422.83

**Table 5:** Average cost on the common successful request set and online decision overhead in the ablation study (R=5000). Runtime is averaged over the three budget settings.

Variant	Cost	Cost	Cost	Runtime
	(*B* = 100)	(*B* = 150)	(*B* = 200)	(ms/req.)
Full PAC–LNS	18.69	23.41	26.66	16.644
w/o PAC priority	18.67	23.49	26.54	17.693
K=1	18.71	23.58	27.26	13.076
w/o LNS	18.70	23.60	26.42	10.864
w/o budget reservation	18.68	23.63	26.83	14.975

**Table 6:** One-factor-at-a-time sensitivity analysis on Alibaba trace replay (R=5000, B=150). Delay and cost are calculated over the family-specific common successful set. Therefore, values should be compared only within the same parameter block.

Parameter	Setting	Default	AR (%)	E2E (ms)	Cost	Time (ms)
Ready-window size *K*	K=1		89.64	1260.74	23.45	8.562
	K=2		89.62	1247.20	23.38	9.981
	K=4	√	89.68	1251.08	23.27	16.478
	K=6		89.54	1262.43	23.41	22.309
	K=8		89.72	1257.94	23.36	24.561
Reservation weight $p_{r}$	$p_{r}=0.00$		88.78	1240.06	23.60	20.243
	$p_{r}=0.05$		89.06	1260.28	23.83	18.967
	$p_{r}=0.10$	√	89.68	1246.77	23.37	20.330
	$p_{r}=0.15$		89.62	1255.76	23.61	20.822
	$p_{r}=0.20$		90.06	1265.38	23.38	21.930
LNS search budget $I_{\max}$	$I_{\max}=0$		89.48	1262.98	23.72	12.339
	$I_{\max}=5$		89.68	1261.23	23.50	20.249
	$I_{\max}=10$	√	89.68	1261.23	23.50	21.236
	$I_{\max}=20$		89.68	1261.23	23.50	14.330
	$I_{\max}=30$		89.68	1261.23	23.50	11.316
Priority scale λ	λ=0.0		89.68	1269.15	23.52	10.867
	λ=1.0	√	89.68	1269.15	23.52	10.873
	λ=2.0		89.68	1269.15	23.52	11.197
Multicast α set	{0}		89.60	1264.36	24.01	7.924
	{0.5}		89.46	1257.89	22.95	7.714
	{1}		89.72	1260.69	22.54	8.175
	{0,0.5,1}		89.68	1253.52	23.17	9.699
	{0,0.25,0.5,0.75,1}	√	89.68	1253.52	23.17	10.917

Note: The check mark (*√*) denotes the default setting of each corresponding parameter.

**Table 7:** Sensitivity to generated dissemination attributes (R=5000, B=150). Each entry reports AR (%)/common-set E2E delay (ms).

Factor	Setting	PAC-LNS	PACP-HEFT	TUL-MPH
Destination ratio	0.3	89.56/1287.78	89.42/1285.64	94.36/2154.78
	0.5	89.98/1269.82	89.50/1290.39	94.26/2121.98
	0.7	89.68/1257.41	89.42/1280.12	94.24/2112.74
	0.9	89.72/1226.80	88.80/1263.74	94.12/2059.89
Result-size model	Baseline	89.68/1257.41	89.42/1280.12	94.24/2112.74
	Uniform	90.00/1277.90	89.40/1262.46	94.20/2112.92
	Lognormal	89.72/1268.80	89.14/1260.07	94.26/2102.32

## Data Availability

The Alibaba Cluster Trace v2018 analyzed in this study is publicly available at https://github.com/alibaba/clusterdata/tree/master/cluster-trace-v2018 (accessed on 9 February 2026). The simulation code and processed data supporting the reported results are available from the corresponding author upon reasonable request.

## Abbreviations

The following abbreviations are used in this manuscript:

DAG	Directed acyclic graph
E2E	End-to-end
FFT	Fast Fourier transform
GE	Gaussian elimination
LNS	Large neighborhood search
PAC-LNS	Priority-Aware Constraint-based Large Neighborhood Search

## References

[1] Xiao H., Xu C., Ma Y., Yang S., Zhong L., Muntean G.M. (2022). Edge intelligence: A computational task offloading scheme for dependent IoT application. IEEE Trans. Wirel. Commun..

[2] Ma M., Wang Z., Guo S., Lu H. (2024). Cloud–edge framework for AoI-efficient data processing in multi-UAV-assisted sensor networks. IEEE Internet Things J..

[3] Gu X., Duan Z., Ye G., Chang Z. (2025). Virtual Node-Driven Cloud–Edge Collaborative Resource Scheduling for Surveillance with Visual Sensors. Sensors.

[4] Umer A., Ali M., Jehangiri A.I., Bilal M., Shuja J. (2024). Multi-objective task-aware offloading and scheduling framework for internet of things logistics. Sensors.

[5] Loconte D., Ieva S., Gramegna F., Bilenchi I., Fasciano C., Pinto A., Loseto G., Scioscia F., Ruta M., Di Sciascio E. (2024). Serverless microservice architecture for cloud-edge intelligence in sensor networks. IEEE Sens. J..

[6] Huang X., Tian B. (2026). Joint Service Chain Orchestration and Computation Offloading via GNN-Based QMIX in Industrial IoT. Sensors.

[7] Zhao G., Xu H., Zhao Y., Qiao C., Huang L. (2021). Offloading tasks with dependency and service caching in mobile edge computing. IEEE Trans. Parallel Distrib. Syst..

[8] Liu L., Tan H., Jiang S.H.C., Han Z., Li X.Y., Huang H. Dependent task placement and scheduling with function configuration in edge computing. Proceedings of the International Symposium on Quality of Service.

[9] Cao Z., Deng X., Yue S., Jiang P., Ren J., Gui J. (2024). Dependent task offloading in edge computing using GNN and deep reinforcement learning. IEEE Internet Things J..

[10] Chen X., Cao J., Sahni Y., Jiang S., Liang Z. (2024). Dynamic task offloading in edge computing based on dependency-aware reinforcement learning. IEEE Trans. Cloud Comput..

[11] Zhu K., Zhang Z., Zeadally S., Sun F. (2024). Learning to optimize workflow scheduling for an edge–cloud computing environment. IEEE Trans. Cloud Comput..

[12] Tian Z., Shi D., Chen Y., Zhao W. (2026). Effective Cloud–Edge Workflow Scheduling via Decoupled Offline Learning and Unified Sequence Modeling. Appl. Sci..

[13] Ma Y., Liang W., Wu J., Xu Z. (2019). Throughput maximization of NFV-enabled multicasting in mobile edge cloud networks. IEEE Trans. Parallel Distrib. Syst..

[14] Ren H., Xu Z., Liang W., Xia Q., Zhou P., Rana O.F., Galis A., Wu G. (2020). Efficient algorithms for delay-aware NFV-enabled multicasting in mobile edge clouds with resource sharing. IEEE Trans. Parallel Distrib. Syst..

[15] Liu Y., Wu Y., Jiao X., Li X., Wu H., Zhou J., Xu Z., Yu X. (2025). Rapid edge-computing for intelligent fiber-optic DAS. IEEE Sens. J..

[16] Li G., Tan H., Liu L., Zhou H., Jiang S.H.C., Han Z., Li X.Y., Chen G. (2023). DAG Scheduling in Mobile Edge Computing. ACM Trans. Sen. Netw..

[17] Lou J., Tang Z., Jia W., Zhao W., Li J. (2023). Startup-aware dependent task scheduling with bandwidth constraints in edge computing. IEEE Trans. Mob. Comput..

[18] Liang Y., Zhang S., Wu J. (2025). Cost-efficient delay-bounded dependent task offloading with service caching at edges. IEEE Trans. Comput..

[19] Zhao L., Zhao Z., Hawbani A., Liu Z., Tan Z., Yu K. (2025). Dynamic caching dependency-aware task offloading in mobile edge computing. IEEE Trans. Comput..

[20] Dong S., Tang J., Abbas K., Hou R., Kamruzzaman J., Rutkowski L., Buyya R. (2024). Task offloading strategies for mobile edge computing: A survey. Comput. Netw..

[21] Barrios C., Kumar M. (2023). Service caching and computation reuse strategies at the edge: A survey. ACM Comput. Surv..

[22] Xie X., Wang H., Liu X. (2023). Scheduling for minimizing the age of information in multisensor multiserver industrial Internet of Things systems. IEEE Trans. Ind. Inform..

[23] Chaudhry R., Tapaswi S., Kumar N. (2019). A green multicast routing algorithm for smart sensor networks in disaster management. IEEE Trans. Green Commun. Netw..

[24] Fan M., Zhao X., Zuo X., Ye L. (2024). A budget-constrained workflow scheduling approach with priority adjustment and critical task optimizing in clouds. IEEE Trans. Autom. Sci. Eng..

[25] Ma X., Gao H., Xu H., Bian M. (2019). An IoT-based task scheduling optimization scheme considering the deadline and cost-aware scientific workflow for cloud computing. EURASIP J. Wirel. Commun. Netw..

[26] Topcuoglu H., Hariri S., Wu M.Y. (2002). Performance-effective and low-complexity task scheduling for heterogeneous computing. IEEE Trans. Parallel Distrib. Syst..

[27] Liu J., Lu S., Che D. A survey of modern scientific workflow scheduling algorithms and systems in the era of big data. Proceedings of the 2020 IEEE International Conference on Services Computing (SCC).

[28] Kou L.T., Markowsky G., Berman L. (1981). A Fast Algorithm for Steiner Trees. Acta Inform..

[29] Pisinger D., Ropke S. (2018). Large neighborhood search. Handbook of Metaheuristics.

[30] Coleman T., Casanova H., Pottier L., Kaushik M., Deelman E., da Silva R.F. (2022). WfCommons: A framework for enabling scientific workflow research and development. Future Gener. Comput. Syst..

[31] Alibaba Cluster Data. https://github.com/alibaba/clusterdata/tree/master/cluster-trace-v2018.

[32] Khan S., Jiangbin Z., Irfan M., Ullah F., Khan S. (2024). An expert system for hybrid edge to cloud computational offloading in heterogeneous MEC–MCC environments. J. Netw. Comput. Appl..

[33] Ma L., Zhang Y., Zhou J., Zhang G. (2024). A gene-inspired metaheuristic for scheduling workflow tasks in mobile edge computing-supported cyber–physical systems. J. Syst. Archit..

[34] Cui B., Zhang J. (2025). Exact and Approximation Algorithms for Task Offloading with Service Caching and Dependency in Mobile Edge Computing. Future Internet.

